# Historical frequency of plants in nursery catalogues predicts likelihood of naturalization in ornamental species

**DOI:** 10.1002/eap.70023

**Published:** 2025-05-11

**Authors:** Thomas N. Dawes, Jennifer L. Bufford, Philip E. Hulme

**Affiliations:** ^1^ Department of Pest‐Management and Conservation Lincoln University Lincoln New Zealand; ^2^ Present address: Manaaki Whenua – Landcare Research Lincoln New Zealand

**Keywords:** boosted classification models, environmental weeds, nursery catalogues, ornamental plants, propagule pressure

## Abstract

Ornamental horticulture is the major pathway of non‐native plant species introductions worldwide. Historic nursery catalogues capture a long‐term view of introduction effort arising from garden plantings and are a powerful resource for understanding why some introduced ornamental species subsequently jump the garden fence. Analyses of historic nursery catalogues can help us understand the reasons for failed invasions as well as why some species successfully naturalize or invade. We used New Zealand nursery catalogues from the 1860s to the 1990s to understand the patterns of failed invasions, as well as successful naturalization and invasion. Together with data on several horticulturally relevant plant traits, we used a boosted classification model to predict the likelihood of naturalization and invasion. A species' maximum height, its frequency in nursery catalogues, and the family‐level global naturalization rate were the most influential variables. Naturalized species were generally taller, more frequently offered for sale, and belonged to families with higher global naturalization rates than those that had not naturalized. Other traits such as cold hardiness or shade tolerance were not significantly different between naturalized and non‐naturalized species but contributed somewhat to the overall model fit. By contrast, our predictions of the likelihood a species would become invasive were poor, without any robust relationships with any of the covariates. This indicates that factors that drive the transition from naturalized to invasive species may be determined by the characteristics of the habitats that they invade. Species incorrectly predicted to be naturalized may not have had sufficient opportunity to do so and may pose a greater risk of naturalizing in the future. This provides an avenue for identifying future naturalized species and enabling proactive management or monitoring of these species of concern.

## INTRODUCTION

The ornamental horticulture industry is one of the most important pathways for the introduction of non‐native plant species into new regions (Dehnen‐Schmutz et al., 2007a; van Kleunen et al., 2018). Globally, approximately 60% of the world's worst plant invaders have resulted from ornamental plantings (Hulme et al., 2018). This is partly attributable to the size of the pool of species available in the ornamental horticulture market. Thousands of plant species are marketed for ornamental horticulture, but often only a small percentage of these ever become naturalized (Hulme, 2012). Thus, an understanding of the role of ornamental horticulture in plant invasions should not simply focus on the few species that become naturalized but should also consider the many species that fail to do so (Diez et al., 2009; van Kleunen et al., 2010).

Ornamental plants may be more likely to invade because species selected for planting in public and private gardens have traits that make them predisposed to “jump the garden fence” (Guo et al., 2019). Horticulturalists generally select for ornamental plants that have traits suited to the introduced environment (van Kleunen et al., 2018). Assessing how species traits, from leaf physiology to reproduction to environmental tolerances, relate to plant invasion success has become an increasingly well‐studied avenue of research (e.g., Gioria et al., 2023; Mathakutha et al., 2019; Nunez‐Mir et al., 2019; Pyšek & Richardson, 2007). Several traits found to facilitate plant naturalization are also often selected by horticulturalists, such as maximum plant height (Pyšek & Richardson, 2007), hardiness to cold temperatures (Maurel et al., 2016), drought tolerance (Li et al., 2023), and vegetative spread (Nunez‐Mir et al., 2019). However, different traits can influence naturalization of persistent populations and invasive impact or spread (Dawson et al., 2009). Understanding how horticultural traits vary in importance across the invasion process can reveal the different drivers of naturalization compared to invasion (Catford et al., 2019; Pyšek et al., 2023). For example, cold hardiness seems to be important for both naturalization and spatial spread of ornamental species planted in botanic gardens in Germany, whereas plant height and growth form increase the likelihood of naturalization but not spatial extent (Hanspach et al., 2008). Thus, understanding how horticultural selection might influence naturalization and invasion would be important for any rapid screening by the industry for future ornamental plant risks.

However, plant traits alone are not sufficient to explain or predict naturalization (Hayes & Barry, 2008; Pyšek & Richardson, 2007). In particular, the interaction between introduction history and plant traits is fundamentally important in determining both naturalization (Bucharova & van Kleunen, 2009) and invasiveness (Kinlock, Dehnen‐Schmutz, et al., 2022; Pyšek et al., 2015). Similarly, the widespread cultivation of a plant species also interacts with traits and introduction history (Guo et al., 2024). Horticultural availability is one of the most important aspects of introduction history (Kinlock, Dehnen‐Schmutz, et al., 2022), as prolonged persistent introduction of a species can lead to a higher introduction effort from gardens (Lockwood et al., 2009). High introduction effort greatly increases the likelihood of naturalization (Catford et al., 2011; Lockwood et al., 2005; Simberloff, 2009), but it can be difficult to quantify. A direct measure of the number of individuals planted in gardens is rarely available and often only possible for relatively small areas (Cubino et al., 2015; Dawson et al., 2011; Mayer et al., 2017). Other measures such as the frequency with which plant species are found in botanic gardens have been used as a proxy for introduction effort at national or regional scales (Hanspach et al., 2008; Hulme, 2011; Ni & Hulme, 2021). However, neither of these measures necessarily captures the fact that the species currently observed as naturalized are the result of planting over many decades. What is currently found in gardens may not reflect historical introduction effort. Records from nursery catalogues may better capture this longer temporal perspective of introduction effort. Historic nursery catalogues provide a valuable source of introduction history, and the frequency with which species occur in horticultural catalogues and nurseries over time has previously been utilized as a proxy for introduction effort (Lavoie et al., 2016; McCulloch‐Jones et al., 2021; Ööpik et al., 2013). Nevertheless, historic nursery catalogues remain an underutilized resource in the study of plant invasions (Bufford et al., 2025b; Dehnen‐Schmutz et al., 2007b; Kinlock, Adams, & van Kleunen, 2022; Lavoie et al., 2016; Pemberton & Liu, 2009), particularly in the southern hemisphere.

While many species are introduced into horticulture in new regions, few establish outside of gardens. Species that fail to naturalize present a key line of evidence that is often ignored in the study of plant invasions (Diez et al., 2009; Zenni & Nuñez, 2013). Comparison of both the traits and introduction history of these failures with those species that have naturalized or become invasive is needed to understand the invasion process as a whole (van Kleunen et al., 2010). The definition of an invasive species in different studies is variable (Richardson et al., 2000) and tends to relate to spatial extent (e.g., Hanspach et al., 2008) or environmental impact (e.g., Ni et al., 2021) or a combination of the two (IPBES, 2023). We describe species as being invasive if they belong to the subset of naturalized non‐native species that are formally listed by the New Zealand Department of Conservation as environmental weeds (sensu Weber, 2017; Williams & West, 2000) due to their perceived ecological impacts on public conservation land (Howell, 2008).

We utilized nursery catalogues and horticultural plant trait resources to build a database of ornamental species introduced to New Zealand gardens and their associated horticulturally relevant traits. Our focus on traits relevant to horticulture aims to replicate the traits utilized by nurseries when deciding which species to cultivate and stock. We used boosted classification models to assess factors that might explain both naturalization and invasiveness (designation as an environmental weed) of ornamental plant species (McGregor et al., 2012; Moodley et al., 2013; Schmidt & Drake, 2011). These models allowed us to assess the relative importance of traits and introduction effort in naturalization and invasion and to ask: do species traits and introduction effort predict (a) ornamental plant species naturalization and (b) whether a naturalized ornamental species will become invasive in natural ecosystems?

## METHODS

### Species selection and nursery catalogue database

To identify the drivers of naturalization and invasion of ornamental plants, we based our initial species selection on those naturalized plant species known to be planted as ornamentals (Gatehouse, 2008) and formally listed as “environmental weeds” by the New Zealand Department of Conservation as naturalized plant species that are perceived as having a significant impact on conservation land (Howell, 2008). This led to an initial list of 328 species, which we subsequently expanded to include all congeners, including present and historic synonyms, using the World Flora Online database (The World Flora Online Consortium, 2023). This resulted in an initial target list of 571 genera that included invasive species (and their historical generic synonyms) as well as their naturalized and non‐naturalized congeners. By comparing congeners, we aimed to account for trait conservatism within genera (e.g., Davies et al., 2013) and the different horticultural histories these genera may have (Kuester et al., 2014). We then searched historical nursery catalogues produced in New Zealand for any species within this extended list of genera.

We used the New Zealand National Nursery Catalogue Collection archived at Lincoln University as the basis for our search since it represents the largest collection of digitized nursery catalogues in the country. We focused on nursery catalogues that sold a wide range of live plants for ornamental purposes. Our search was initially focused on the handful of nursery catalogues published between 1861 and 1891 in order to better capture the earliest introduction dates. Subsequently, as the horticultural market grew in New Zealand and more nursery catalogues were published, we selected one catalogue approximately every 5 years from 1895 to 1992 (giving a total of 29 nursery catalogues) to get a broad temporal sample. From 1925 onward, we focused on the catalogues issued by the Duncan & Davies nursery since it was one of the longest‐operating and largest ornamental nurseries in New Zealand, selling a wide range of plant species directly to consumers. Duncan & Davies also imported plants from overseas (Jellyman, 2011). We therefore assumed the Duncan & Davies nursery would be representative of the national horticultural market while also likely influencing gardening trends in New Zealand. A unique feature of our analysis is that it covers the growth of the horticultural market from a point when there were only 100,000 inhabitants in New Zealand in 1860 to 3.5 million in 1992 (Briggs, 2003). Over this period, which predates online sales, nursery catalogues were distributed by post throughout the country, thus regional variation in the location of a specific nursery is unlikely to be a confounding factor. The text from each digitized catalogue was extracted using Amazon Textract (aws.amazon.com/textract), a commercial machine learning service for optical character recognition that enabled us to automate catalogue processing to extract species names. These data were manually checked and corrected. All species names extracted from the catalogues were updated to their current binomial using the World Flora Online database (The World Flora Online Consortium, 2023) and merged with the data on non‐native species status in the naturalized flora of New Zealand database (Brandt et al., 2020, 2021). In doing so, we retained all well‐known hybrid taxa that could be linked to an unambiguous name as many hybrids can become naturalized. Difficult or unnamed hybrid taxa such as hybrid *Rhododendron* were not kept in the dataset. Cultivars and other infraspecific taxa were pooled at the species level. This resulted in a species database that included known ornamental species, their frequency in different catalogues, and their status as either naturalized or invasive species. We used the total number of catalogues that a species occurred in (catalogue frequency) as a measure of the historical prevalence of a species in the horticultural industry, which was our proxy for introduction effort. Searched species not found in any catalogues were not included in our database, and thus catalogue frequency could not be zero. Catalogue frequency encompasses several dimensions of introduction effort as it was correlated with the earliest year a species was recorded in the catalogues (Spearman's rank correlation coefficient = −0.45, df = 954, *p* < 0.001, Appendix S1: Figure S1) as well as the range of years spanned in the catalogues (latest year minus earliest year; Spearman's rank correlation coefficient = 0.93, df = 954, *p* < 0.001, Appendix S1: Figure S2).

### Trait database collation

In order to determine the role of plant traits in naturalization and invasion, we compiled data from horticultural databases. The selection of plant traits was based on two criteria: (a) their likely use by gardeners or horticulturalists in selecting species for planting and (b) their potential to influence naturalization or invasiveness. This led to a selection of 12 traits (Table 1). Although we initially considered including plant life history, fewer than 10 species in our target list were annuals, and thus, we omitted these species and focused our analyses solely on perennials. The primary sources used to collate plant traits were Dave's Garden (DG, https://davesgarden.com/guides/pf/, accessed August 2022), the National Gardening Association of the United States (NGA, https://garden.org/, accessed on 24th November 2022), and the USDA plants Traits Database (USDA NRCS, 2023). While these data sources provided most of our trait data, gaps were filled by checking additional sources that provided reliable trait measurements consistent with those obtained for other species in the database (Appendix S2) or by generalizing at the genus level in a very small minority of cases for appropriately conserved traits. In some cases, it was not possible to obtain all trait data for a species, and thus, only species that had three or fewer missing trait values were retained for analysis.

**TABLE 1:** Expectations regarding the traits used in our analyses.

Trait	Expectation
Habit	Trees/shrubs may be more likely to naturalize and become invasive than herbs (Hanspach et al., 2008). Climbers may also be more likely to become invasive than herbs or trees (Lavoie et al., 2016) due to their ease of vegetative spread
Max height	Taller species may be more likely to naturalize and become invasive (Hanspach et al., 2008, Pyšek & Richardson, 2007)
Soil pH range	Species that can tolerate a broader range of soil pH conditions may be more likely to naturalize and become invasive (Wamelink et al., 2018)
Minimum temperature tolerated	Species that tolerate lower minimum temperatures may be hardier to New Zealand climate and thus more likely to naturalize and become invasive (Hanspach et al., 2008, Maurel et al., 2016)
Deciduousness	Evergreen species may be more likely to naturalize and become invasive in New Zealand than deciduous species (Bucharova & van Kleunen, 2009) because of the relative scarcity of deciduous species in the New Zealand flora (McGlone et al., 2004)
Dry soil tolerant	Species that can grow in dry or drought‐like conditions may be better suited to naturalization or becoming invasive (Li et al., 2023)
Wet soil tolerant	Wet‐soil‐tolerant species may be better suited to New Zealand's high rainfall and high humidity climate and therefore more likely to naturalize
Sun tolerant	Species that can tolerate full sun conditions may be better able to establish in open habitat and thus more able to naturalize (Sutherland, 2004)
Shade tolerant	Shade‐tolerant species may be better able to invade intact forest and thus be listed as environmental weeds (Fridley et al., 2021)
Propagated by seed	Capability to reproduce by seed (and thus likely propagation by seed) increases invasion success (Küster et al., 2008)
Propagated vegetatively	Species propagated vegetatively may be better able to naturalize (Lloret et al., 2005, Thomas et al., 2012)
Propagated by roots	Species propagated by root cuttings or division may be more likely to naturalize or spread due to the movement of soil

*Note*: The expectations are drawn from a variety of published sources. Habit is a categorical trait with three groups (shrubs/trees, herbaceous plants, climbers). Maximum plant height, soil pH range, and minimum temperature are continuous variables. The remaining traits are binary. For soil moisture and shade tolerance as well as propagation methods, these binary traits are not mutually exclusive, and thus, a species that can grow in a wide range of light conditions would be scored as 1 for both shade and sun tolerance.

To account for taxonomic propensity to naturalize, we calculated the proportion of species in each family that had naturalized globally for each family represented in our dataset. We used the GloNAF database (van Kleunen et al., 2019) to calculate the total number of species in each family that have naturalized anywhere in the world (following Pyšek et al., 2017). Using the WFO database (The World Flora Online Consortium, 2023), we extracted the total known number of species in each of these families. We removed varieties and subspecies, but retained hybrids, matching the approach used in our species‐trait database, and checked for consistent nomenclature across databases. The proportion of naturalized species out of the total species in that family was calculated as a measure of taxonomic naturalization propensity (following Hulme & Liu, 2022; Wyse & Dickie, 2017).

This left a final species‐trait matrix for our naturalization model containing 956 species (899 with complete data) across 12 traits, along with catalogue frequency, family naturalization rate, and both naturalization and invasion status. Approximately one third of the species were naturalized (338), of which 178 were listed as invasive. Data are archived on FigShare (DOI: 10.6084/m9.figshare.28348340).

### Statistical analyses

Many of our traits were correlated with each other, and the multiple binary variables covering one trait were also often non‐independent. For example, habit and deciduousness were associated, with trees and shrubs more likely evergreen than expected by chance and with climbers more likely deciduous compared to chance expectations (χ^2^ = 8.29, *p* = 0.016). Because of these non‐independence issues in the data matrix, we used a boosted classification tree model, a machine learning technique that combines information from multiple “weak learner” trees into a single predictive model (Elith et al., 2008). Boosted classification tree approaches can model non‐independent data, handle multiple interactions between variables, and are robust to missing values (Buston & Elith, 2011). Thus, we built two boosted classification models: first to classify differences between non‐naturalized and naturalized ornamental plants (including invasive species), and then within the subset of naturalized species, to classify those species that were or were not classed as invasive. We used the gradient boosting algorithm for both the naturalization and invasiveness models from the “gbm” R package (Ridgeway, 2020).

Prior to running both models, all continuous and ordinal data were rescaled to a standardized/Z‐distributed scale with a mean of zero. A random 80% of the dataset was used as training data to build the model, with the remaining 20% set aside as model testing data. We also ran each model with 10‐fold cross‐validation of the testing data to avoid overfitting. The learning rate was set to 0.001 to allow slow reliable convergence on an optimal model, while a tree complexity of 5 was chosen to allow for multiple interactions between traits. The minimum number of observations in the terminal nodes of a tree was set to 10 and the bag fraction to 0.5—the default values for the gbm model. The model was initially run with the number of trees (nt) set from 1000 to 20,000, and the function “gbm.perf” was used on these runs to select a value for nt that minimized predictive deviance (Elith et al., 2008). The optimum number of trees was identified as 8600 for the naturalization model and 1000 for the invasiveness model. Lastly, in order to capture the variability in model output for a stochastic machine learning technique, we ran the final model 100 times with a different random seed and different training sample (following Yang et al., 2016) and used mean model metrics across the 100 runs of the models.

We used the area under the receiver operator characteristic curve (AUC) and the Mathews correlation coefficient (MCC) to assess model fit, given that these metrics were robust to the fact that naturalized and non‐naturalized species were not of equal prevalence (Chicco & Jurman, 2020). The MCC is the most appropriate metric for binary response variables (Chicco & Jurman, 2020), especially with regard to both sensitivity and specificity (Chicco & Jurman, 2023). We interpreted AUC scores above 0.9 as excellent models, while values below 0.7 were considered poor (Carter et al., 2016; Hanley & McNeil, 1982). Since the MCC score is a contingency matrix method of calculating the Pearson product–moment correlation coefficient (Powers, 2011), it can be interpreted in the same way, with values >0.40 reflecting a strong positive relationship, while values <0.20 have no relationship or a negligible relationship (Chicco & Jurman, 2020). For calculating MCC scores, a cutoff value of 0.5 was used to convert the probabilities produced by the model into a binary classification as per convention (Chicco & Jurman, 2023). We carried out Kruskal–Wallis tests to assess differences in species catalogue frequency, maximum height, and their family global naturalization rate in relation to species status using Dunn's test for post hoc pairwise comparisons.

All data processing, analyses, and figure preparation were carried out in R (R Core Team, 2020), using the R Studio interface (RStudio Team, 2016). The “caret” package (Kuhn, 2015) was used for data partitioning, the “gbm” package (Ridgeway, 2020) was used to run the models, and the “ModelMetrics” package (Hunt, 2020) was used to calculate AUC and MCC metrics. The “ggplot2” package (Wickham, 2016) was used for plotting figures.

## RESULTS

**FIGURE 1:**
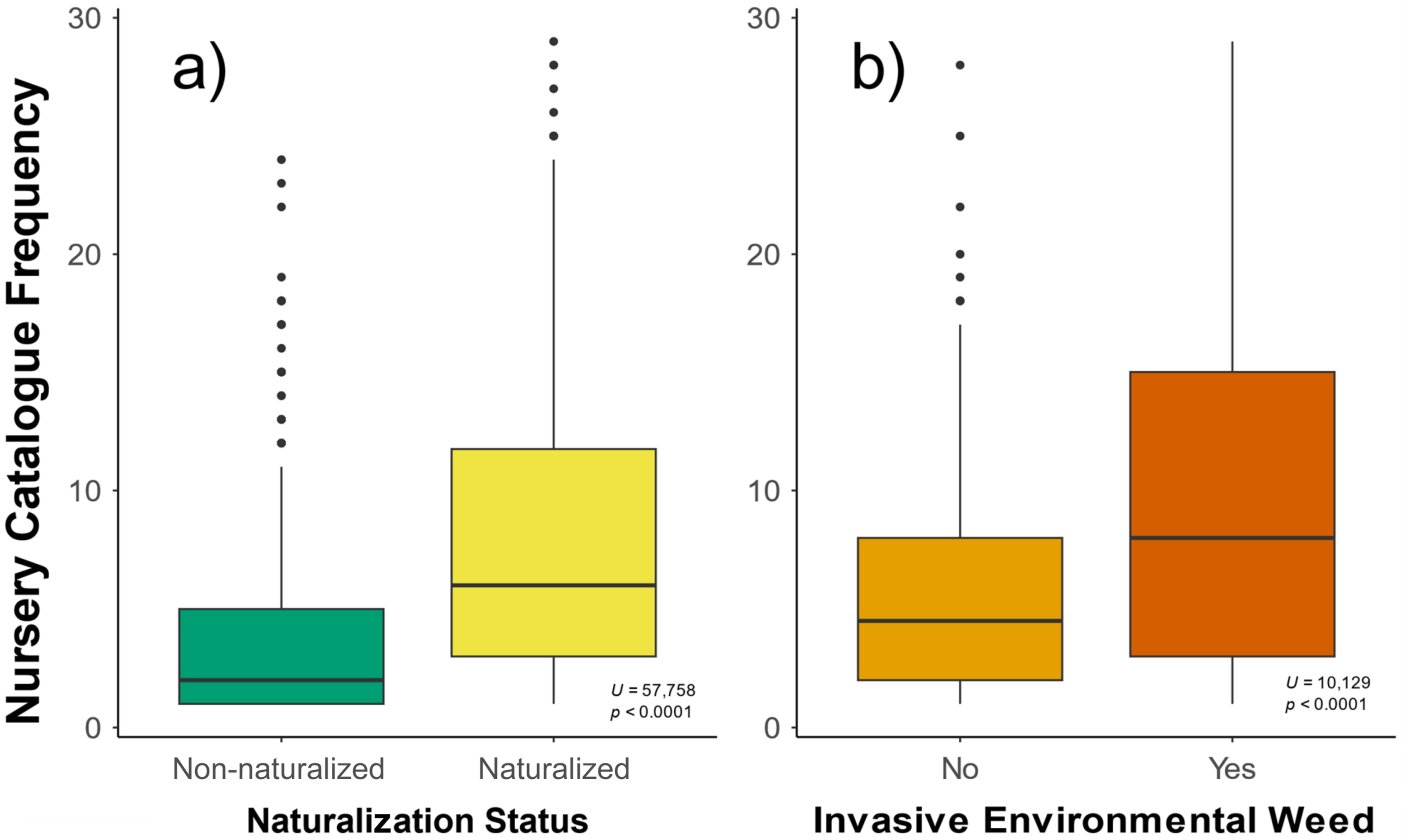
Difference between (a) non‐naturalized species (shown in green) and naturalized species (shown in yellow) in their historic nursery catalogue frequency and (b) species listed as invasive species (dark orange) and naturalized species that are not invasive (light orange). The result and statistical significance of a post‐hoc Mann‐Whitney U test are shown on the bottom right of each panel.

Species in our dataset were more likely to be trees or shrubs than climbers or herbaceous species and more likely evergreen than deciduous (Table 2). There was a marginal statistical difference in the distribution of habit among the non‐naturalized, naturalized, or invasive species (χ^2^ = 9.29, *p* = 0.054, Table 2). Rosaceae and Fabaceae were the two largest families in the overall dataset and the most abundant of both the naturalized and invasive species. Invasive species had a greater median nursery catalogue frequency than either other naturalized species or non‐naturalized species (Table 3; Figure 1a). Maximum height (Table 3, *p* = 0.016) was higher in invasive species, and the proportion of the family naturalized globally (Table 3, *p* = 0.007) was higher for naturalized species.

**TABLE 2:** The total number of species in each combination of habit, deciduousness, naturalization, and invasive status.

			Naturalized species			
	Non‐naturalized		Not invasive		Invasive	
	Evergreen	Deciduous	Evergreen	Deciduous	Evergreen	Deciduous
Trees/shrubs	247	193	60	47	83	35
Herbaceous	61	47	19	22	21	16
Climbers	30	37	4	5	10	13
Total	338	277	83	74	114	64

*Note*: Herbaceous perennials classified as “deciduous” can include plants that dieback to a bulb or lose their vegetation at a specified period of year. Note that six species have missing data for deciduousness and are therefore omitted from this table.

**TABLE 3:** Median values for three key numeric variables in our dataset by each naturalization and invasive environmental weed status.

		Naturalized species		
	Non‐naturalized	Not invasive	Invasive	Kruskal–Wallis
Catalogue frequency	2^a^	4.5^b^	8^c^	151.79, *p* < 0.001
Maximum height	3.6^a^	3^a^	5^b^	9.34, *p* = 0.009
Family naturalization rate	0.0434^a^	0.0517^b^	0.0515^b^	7.35, *p* = 0.025

*Note*: Kruskal–Wallis tests compare the three invasion statuses; each Kruskal–Wallis test has 2 df. The results of pairwise Dunn's tests are indicated by superscript letterings under those columns, with different letters between any two invasion statuses indicating significantly different values at an alpha of 0.05.

**FIGURE 2:**
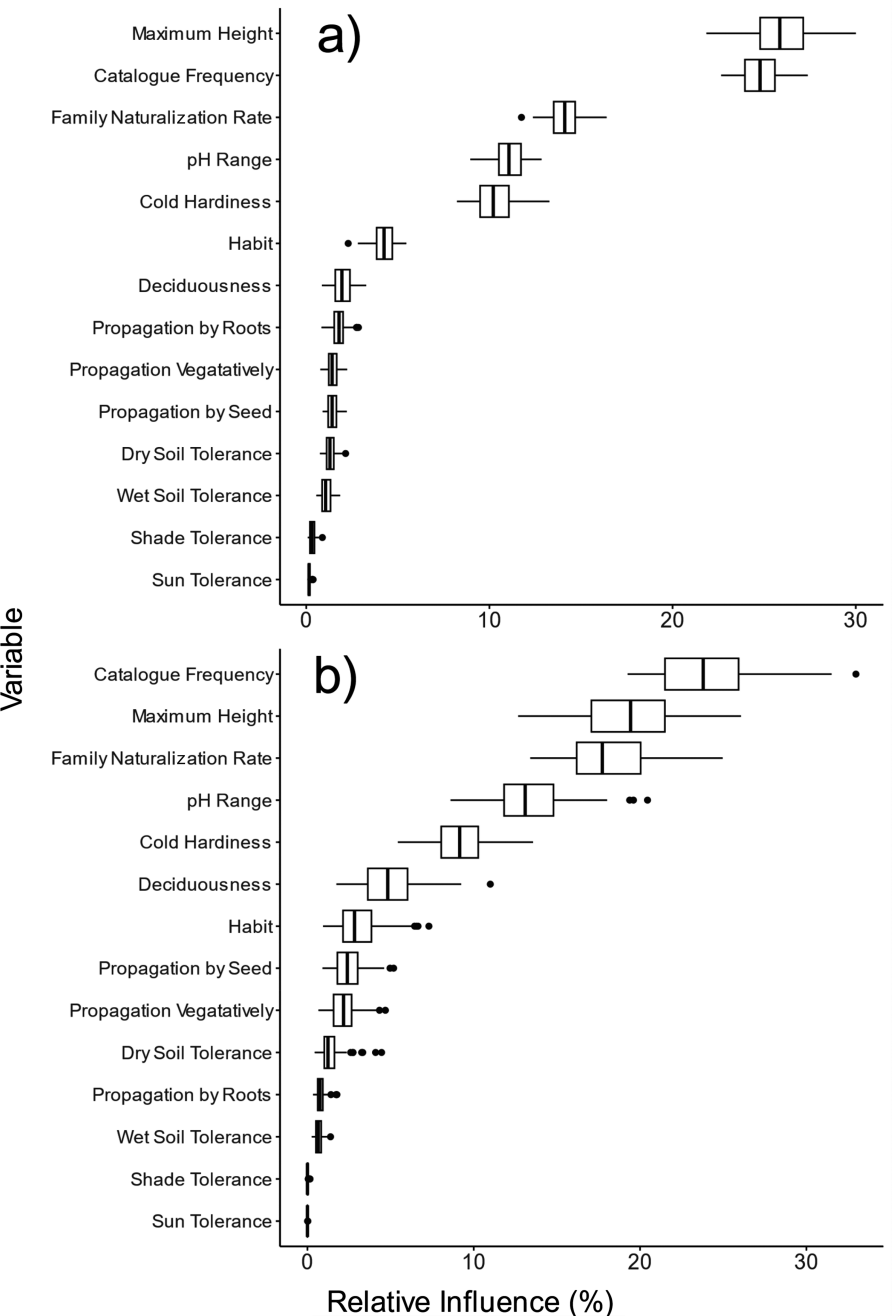
Boxplots showing the relative importance of each variable across 100 runs of the boosted classification tree models for (a) naturalization and (b) likelihood of becoming an environmental weed. Note that each model run has variable influence which sums up to 100%. The boxplots demonstrate the variation in results across the 100 machine learning runs, each with a randomized starting seed. The naturalization model (a) is more consistent across 100 runs than the invasiveness model (b), indicated by the narrower spread of outcomes.

For our naturalization analysis, the mean model (averaged over 100 runs) correctly classified 83% of the species (visualized in Appendix S3: Figure S1). The model had an AUC score of 0.80 and an MCC score of 0.42, which both indicate good predictive power. Three variables accounted for the majority of the discriminatory power of the model: maximum plant height (26% mean relative influence), catalogue frequency (25%), and family naturalization rate (14%), with other traits having relatively little influence (Figure 2a). Beyond the three most influential variables, pH range (11%) and the minimum temperature tolerated (10%) were the next most important variables in the model. The naturalization model incorrectly predicted 46 (5%) species as naturalized (Appendix S4). These species may be considered a possible risk of naturalization, especially those that have multiple congeners that are already naturalized in New Zealand and have a similar suite of traits, such as *Prunus* and *Agapanthus* (Appendix S4: Table S1). The model also incorrectly predicted 114 (12%) naturalized species as non‐naturalized, such as *Ricinus communis* (Appendix S4: Table S2).

The invasiveness model correctly classified 74.5% of species (visualized in Appendix S3: Figure S2). The mean AUC score of 0.63 and an MCC score of 0.20 both indicate this model had relatively weak predictive power. Similar to the naturalization model, catalogue frequency (25% mean relative influence), maximum plant height (20%), and family naturalization rate (18%) were the most important variables (Figure 2b). Nursery catalogue frequency was higher in species designated as invasive (Table 3, *p* < 0.0001; Figure 1b). The invasiveness model incorrectly predicted 41 (12%) species as invasive species, with *Cupressus sempervirens* and *Juglans regia* as the species with the highest predicted probability of being invasive species, despite not having been listed as environmental weeds by the New Zealand Department of Conservation (Appendix S5).

## DISCUSSION

Our results show that, on average, naturalized species (including invasive species) are taller than non‐naturalized species and have a higher frequency in nursery catalogues. These species are also more likely to come from a family with a higher global propensity to naturalize. The other horticultural traits we examined did not differ markedly between naturalized and non‐naturalized species but did contribute to the naturalization model, which had a good discriminatory power to distinguish between naturalized and non‐naturalized species. Conversely, our model predicting those naturalized species that become invasive had a lower discriminatory power and poor model metrics. The difference between these two models indicates that naturalization and invasion, as defined by impact, are driven by different traits and processes and that common horticultural traits better predict naturalization than invasiveness.

The identification of traits that discriminate between naturalized and non‐naturalized plant species can often be confounded by introduction biases that skew the trait distributions toward certain values (Chrobock et al., 2011; Omer et al., 2021). Such biases are particularly common with ornamental plant introductions (Dong et al., 2023), where selection for particular traits, such as plant height (Maurel et al., 2016), or specific plant families, such as the Asteraceae (Diez et al., 2009), by horticulturalists can lead to overrepresentation of certain traits among the pool of naturalized species (Gioria et al., 2023). For this reason, studies need to identify the appropriate species pool from which naturalized species are drawn and account not only for the traits of those species that naturalize but also for those that fail (van Kleunen et al., 2010). By comparing closely related species from the same introduced species pool that either have or have not naturalized, our analysis accounts for introduction bias, giving us greater confidence that these traits are actually associated with the naturalization process.

Species maximum plant height was the most important plant trait in our naturalization model, which is consistent with previous studies that have found greater maximum height in naturalized species than in non‐naturalized species (Goodwin et al., 1999; Hanspach et al., 2008; Pyšek & Richardson, 2007). Maximum plant height is often considered a proxy for the ability to compete for light (Mahaut et al., 2023) and is often interpreted as indicating that stronger competitors are more likely to naturalize. Plant height is confounded with life‐form, with shrubs and trees generally taller than herbaceous perennials. However, our inclusion of life‐form (habit) in the model accounted for this, and habit was only of limited importance in the naturalization model.

The importance of catalogue frequency in our naturalization model is reflective of the significance of introduction effort in determining plant naturalization. The pattern of higher introduction effort leading to higher naturalization rates is widely accepted (Colautti et al., 2006; Lockwood et al., 2009; Pyšek et al., 2015; Simberloff, 2009), although it does not always play a role (Nuñez et al., 2011). High introduction effort increases the probability that a non‐native plant species will find opportunities to become established, such as a suitable microclimate, an empty ecological niche, or a microhabitat free from natural enemies, and can accelerate range expansion (Gioria et al., 2023). Our nursery catalogues offer a long‐term view of how consistently species are being offered for sale, and therefore planted, across over a century. This means our metric captures a broad historical perspective, even if it is an indirect proxy of the number of plants sold. The results reiterate the importance of the role of the horticultural market in driving naturalization of non‐native species (van Kleunen et al., 2018) and are consistent with the idea that introduction history may be more important than most individual plant traits (Blackburn et al., 2013; Colautti et al., 2006; Dawson et al., 2011). This means that even plants currently considered unlikely to naturalize, if promoted heavily and planted widely, could have an increasing risk of naturalization. Weed risk assessments based solely on life‐history traits, therefore, may underestimate the risk of future naturalizations. A lack of evidence of failures and low accuracy hamper weed risk assessments (Hulme, 2012), and this reemphasizes the importance of examining those species that fail to naturalize and accounting for introduction history in these assessments. The limited explanatory power of several plant traits may be because taxonomic affinity better captures a suite of relevant plant traits, as reflected in the importance of our family variable. There is a phylogenetic propensity to naturalize (Omer et al., 2022) and become invasive (Qian, 2023; Qian & Sandel, 2023), with certain genera and families identified as more likely to occur in local alien floras (Pyšek, 1998, e.g., Poaceae, Brassicaceae) and to naturalize (Kuester et al., 2014, e.g., Polygonum, Solanum).

Although post hoc testing showed that there were no marked differences between naturalized and non‐naturalized species in either soil pH range or minimum temperature hardiness, these horticultural traits both contributed modestly to the model indicating that these factors may play a role in naturalization. Cold hardiness has been identified as a significant trait in studies of plant naturalization in the temperate northern hemisphere (Hanspach et al., 2008; Lavoie et al., 2016; Maurel et al., 2016). In contrast, soil pH range has rarely been identified as a significant trait (but see Wamelink et al., 2018), and the role of soil pH range in determining naturalization remains to be understood.

Other traits contributed little to the overall model, despite our expectations (Table 1). Habit was marginally more important than the other categorical plant traits in our model but did not support studies which have indicated habit significantly affected naturalization (Bucharova & van Kleunen, 2009; Hanspach et al., 2008; Sutherland, 2004). Deciduousness is a trait that has rarely been implicated in naturalization (Bucharova & van Kleunen, 2009), so it is perhaps not surprising that we did not find it to be important. Likewise, the binary categorizations of propagation methods, sun tolerance, and soil moisture tolerance lacked predictive power potentially because our categorizations may be too broad to yield meaningful differences. Drought and wet soil tolerance are likely to be important in colonizing some habitats, but not all. Studies indicating that invasive non‐native species exhibit greater drought tolerance as important are also relatively scarce (Li et al., 2023).

Our focus on horticulturally relevant traits captures the responsiveness of plant to different garden environments but means that physiological or genetic traits that could be important were not examined. For example, a small genome has been found to be an advantage during naturalization, being linked to traits favoring adaptation to local conditions (Guo et al., 2024; Pyšek et al., 2023). Climate matching has proven useful in the analysis of naturalization across continental scales (Dong et al., 2023) and even at the smaller spatial scale of New Zealand (Gravuer et al., 2008; McGregor et al., 2012). In addition, plant species with native relatives have been found more likely to naturalize in New Zealand (Diez et al., 2009). Thus, the incorporation of climate matching, genome size, and data on native conspecifics into the analysis may have improved model fit, but many of these variables are themselves proxies for other traits and could reasonably have been captured by our inclusion of environmental tolerances, growth form, and family propensity to naturalize. Therefore, including additional traits would be unlikely to affect our conclusions regarding the importance of introduction effort in plant naturalization in New Zealand (Gravuer et al., 2008; McGregor et al., 2012).

Invasiveness of ornamental plants was not well predicted by catalogue frequency and plant traits. There is likely to be a genuine difference between the importance of traits in driving naturalization and invasiveness, with traits and introduction effort playing a clearer role in driving naturalization (Catford et al., 2019; Dawson et al., 2009; Hanspach et al., 2008). For example, Hanspach et al. (2008) indicated that only cold hardiness is important for invasiveness, while hardiness along with a range of other traits played a role in naturalization. Furthermore, some traits that increase naturalization success (e.g., genome size) may become disadvantageous with regard to invasion extent (Pyšek et al., 2023). However, differences in how invasion is defined can significantly impact which traits are associated with “invasiveness” (Catford et al., 2016). Our designated invaders are exclusively species that impact conservation land (Howell, 2008), while other studies have used spatial extent or spread rate, which are likely to be driven by a different set of traits (Palma et al., 2021). Given our emphasis on impact in conservation land, the traits that make a species invasive will likely differ by habitat invaded (e.g., shade tolerance in podocarp forest vs. cold hardiness in open alpine grasslands) or may be driven by interactions with native species, which can determine impact. This may explain why patterns in invasion were more difficult to detect in our study compared to naturalization, which is only dependent on the ability to reproduce in any of the available habitats (Fridley et al., 2021).

The species incorrectly predicted as naturalized (Appendix S4: Figure S1) should not be ignored; they may even represent species at a high risk of naturalizing in the future. The model indicates that these species have traits suitable for naturalization, even if this naturalization potential has yet to be realized. For example, all the top 5 misclassified species (Appendix S4: Figure S1) are naturalized in multiple parts of the world (GloNAF database, van Kleunen et al., 2019). Therefore, these species may represent an invasion debt (Essl et al., 2011) and may naturalize in the future. Additionally, two of the five species have congeners naturalized within New Zealand. It thus makes sense to target these species with preventative management or policy, such as sale restrictions. For example, the National Pest Plant Accord (NPPA, 2020) prohibits certain species from sale yet does not currently cover all the species considered invasive in New Zealand, offering further scope for regulation in this area (Hulme, 2020). Being misclassified by our model could indicate that these species are candidates for further risk assessment, closer monitoring, or additional regulation under a precautionary approach.

On the other hand, several species that have become naturalized were misclassified as non‐naturalized (Appendix S4: Figure S2). This may be because our measure of introduction effort based on catalogue frequency did not adequately capture all sources of species introduction. For example, forestry has been responsible for the large‐scale planting of several species also sold through ornamental horticultural catalogues, for example, pines (McGregor et al., 2012) and acacias (Hulme, 2023). Indeed, all five naturalized species with the lowest predicted probability of being naturalized (Appendix S4: Figure S2) have a catalogue frequency of only 1 or 2, indicating a low introduction effort through the horticultural nursery trade. Our focus on the live‐plant trade may also have underestimated horticultural sales through catalogues of seeds, bulbs, fruit trees, or taxon‐specific marketing.

## CONCLUSIONS

Predicting which ornamental species may naturalize or become invasive is an important component of managing plant invasion risks (Hulme, 2012). We showed that a knowledge of the frequency with which plant species were presented in nursery catalogues over more than 100 years was an important variable in determining the risk of subsequent naturalization. Indeed, our models have likely highlighted several species that have yet to naturalize in New Zealand but have the potential to do so, potentially informing future regulations regarding the sale of these species. Although there was also a link between nursery catalogue frequency and the likelihood that a species might become invasive, this relationship was less strong. This highlights the complex nature of non‐native species impacts, including how impacts are assessed, and emphasizes that different traits and processes drive naturalization and invasion. Nursery catalogue data are likely available for many countries, and our recommendation is that greater use of this resource should be made to inform weed risk assessments.

## AUTHOR CONTRIBUTIONS

Philip E. Hulme obtained the funding. Thomas N. Dawes, Jennifer L. Bufford, and Philip E. Hulme planned and designed the research. Jennifer L. Bufford and Thomas N. Dawes collected and processed the data. Thomas N. Dawes analyzed the data. Thomas N. Dawes wrote the initial draft of the manuscript, with extensive contributions from Philip E. Hulme and Jennifer L. Bufford. All authors contributed to editing and revising the manuscript.

## CONFLICT OF INTEREST STATEMENT

The authors declare no conflicts of interest.

## Supporting information


Appendix S1.

Appendix S2.

Appendix S3.

Appendix S4.

Appendix S5.

## Data Availability

Data and code (Bufford et al., 2025a) are available in Figshare at https://doi.org/10.6084/m9.figshare.28348340.
