# Supplementary material for: Historical frequency of plants in nursery catalogues predicts likelihood of naturalization in ornamental species
Source: Ecol Appl. 2025 May 11;35(3):e70023. doi: 10.1002/eap.70023 (PMC12066803; doi:10.1002/eap.70023)
Supplement: Supplementary file 1 — Appendix S1. [file EAP-35-e70023-s003.pdf]

Historical frequency of plants in nursery catalogues predicts likelihood of naturalization in ornamental species. Thomas N. Dawes, Jennifer L. Bufford, and Philip E. Hulme. *Ecological Applications*.

<u>Appendix S1</u>

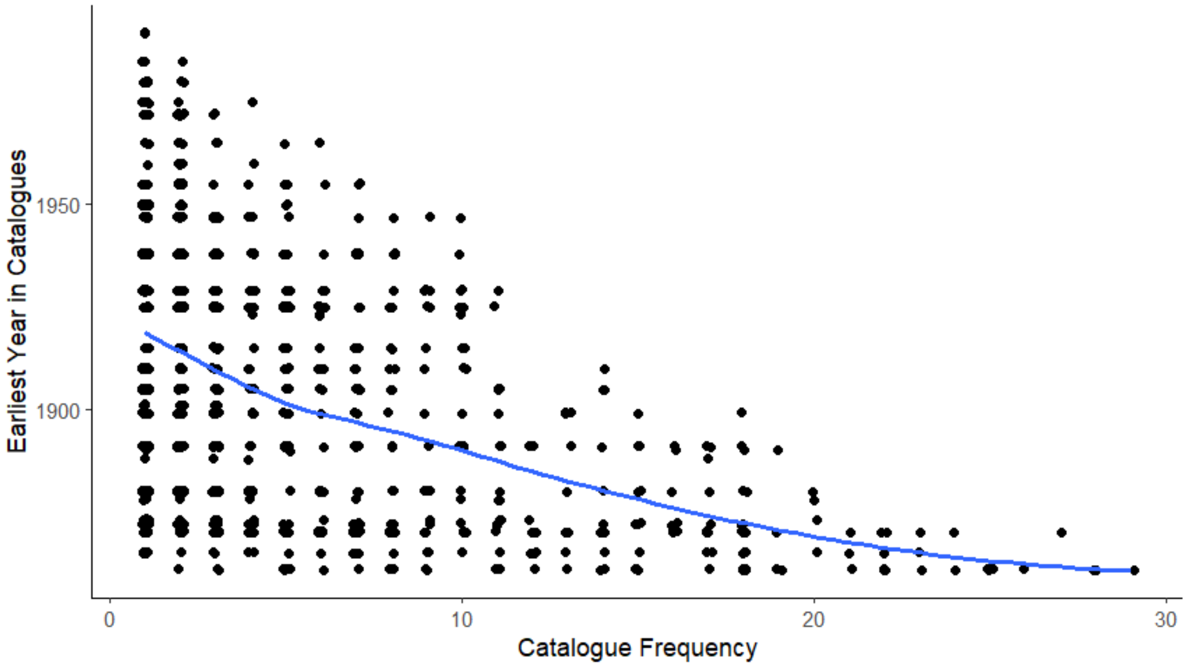

Appendix S1: Figure S1 – A plot showing the correlation between the number of catalogues species appeared in (catalogue frequency, x-axis) and year of the earliest catalogue that species appeared in. A loess curve has been fitted to show the correlation.

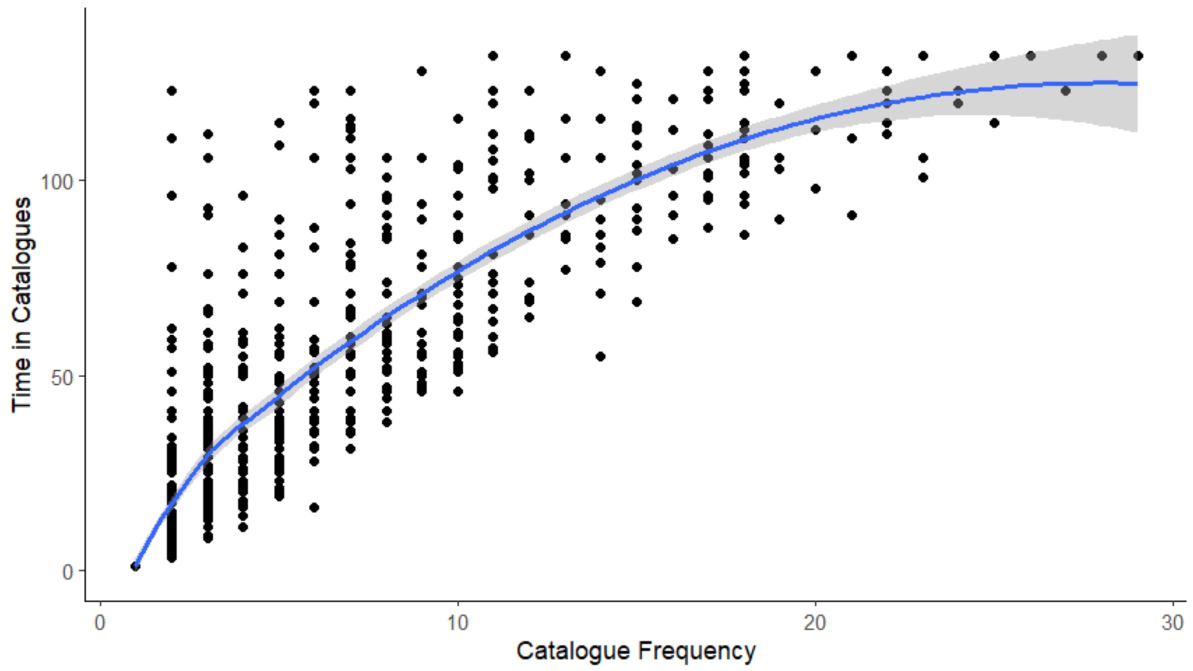

Appendix S1: Figure S2 – A plot showing the correlation between the number of catalogues species appeared in (catalogue frequency, x-axis) and the range of time in catalogues of those species – latest year recorded minus earliest year recorded (y-axis). A loess curve has been fitted to show the correlation.
