# Supplementary material for: Historical frequency of plants in nursery catalogues predicts likelihood of naturalization in ornamental species
Source: Ecol Appl. 2025 May 11;35(3):e70023. doi: 10.1002/eap.70023 (PMC12066803; doi:10.1002/eap.70023)
Supplement: Supplementary file 2 — Appendix S2. [file EAP-35-e70023-s002.pdf]

Historical frequency of plants in nursery catalogues predicts likelihood of naturalization in ornamental species. Thomas N. Dawes, Jennifer L. Bufford, and Philip E. Hulme. *Ecological Applications*.

Appendix S2

Table S1: Tables listing the sources used for the manual data filling. The tables are divided by the frequency of use. Some sources were used often, others were used once only, for example for a specific statement or number.

Source Name	Source - URL
**(A) Common/Frequently-used sources**	
Missouri Botanical Garden	www.missouribotanicalgarden.org
Plants For A Future	pfaf.org/user
North Carolina Plant Toolbox	plants.ces.ncsu.edu
Royal Horticultural Society	www.rhs.org.uk
PlantZAfrica	pza.sanbi.org
Shoot Gardening	www.shootgardening.com
Backyard Gardener	www.backyardgardener.com
Hortipedia	en.hortipedia.com
**(B) Moderately-used sources**	
New Zealand Plant Conservation Network	nzpcn.org.nz
Plant This	plantthis.co.nz / plantthis.com.au
Garden Tags	gardentags.com
Trees and Shrubs Online	www.treesandshrubsonline.org
Gardeners World	www.gardenersworld.com
Useful Tropical Plants Database	tropical.theferns.info
Useful Temperate Plants Database	temperate.theferns.info
Gardening with Angus	www.gardeningwithangus.com.au
Gardens Online	www.gardensonline.com.au
Plant Care Today	plantcaretoday.com
Australian Plants Society NSW	resources.austplants.com.au
World of Succulents	worldofsucculents.com
Practical Plants	practicalplants.org
Picture This	www.picturethisai.com
the Spruce	www.thespruce.com
American Conifer Society	conifersociety.org
Conifers Garden	conifersgarden.com
The Gymnosperm Database	www.conifers.org
Auckland Botanical Gardens	www.aucklandbotanicgardens.co.nz
New Zealand Flora	www.nzflora.info
Earth and Jungle	www.earthandjungle.com
CABI Digital Library	www.cabidigitallibrary.org
Las Pilitas Native Plant Nursery	www.laspilitas.com
**(C) Single/Infrequent Use Sources**	
Silverhill Seeds and Books	silverhillseeds.co.za

FFL Butterfly Gardens	ffl.ifas.ufl.edu/butterflies
Ethiopia Sheep and Goat Productivity Improvement Program	http://esgpip.langston.edu/
Feedipedia	www.feedipedia.org
Calscape (California Native Plant Society)	calscape.org
Best Plants	bestplants.com
Global Flowers	global.flowers/en
Soil, Seed and Garden	soilseedandgarden.com
Garden Guides	www.gardenguides.com
Daleys Fruit	www.daleysfruit.com.au
Yarra Ranges Council - Local Plant Directory	www.yarraranges.vic.gov.au/PlantDirectory
Southern Woods Nursery	www.southernwoods.co.nz
Logee's - Fruiting, Rare and Tropical Plants	www.logees.com
Australian Native Plants Society	anpsa.org.au
Planet Natural	www.planetnatural.com
Ballyrobert Gardens	www.ballyrobertgardens.com
Consulta Plantas	consultaplantas.com
Illinois Wildflowers	www.illinoiswildflowers.info
Australian Seed	www.australianseed.com
The Original Garden	theoriginalgarden.com
Sequoia Valley Farms	www.sequoiavalleyfarms.com.au
Australian National Botanic Gardens	www.anbg.gov.au
Annie's Annuals and Perennials	www.anniesannuals.com
Woody Plants Database (Cornell University)	woodyplants.cals.cornell.edu
Garden Chronicle	gardenchronicle.com
Invasive Plant Atlas of the United States	www.invasiveplantatlas.org
US Forest Service - Fire Effects information System (FEIS)	www.feis-crs.org/feis/
Plant Lust	plantlust.com
Trade Winds Fruit	www.tradewindsfruit.com
Davis Landscape Architecture	davisla.wordpress.com
Plant Info	plantinfo.co.za
Yara	www.yara.com.au
My Mediterranean Garden	mymediterraneangarden.com
Global Invasive Species Database - Invasive Species Specialist Group	iucngisd.org
Wisconsin Horticulture	hort.extension.wisc.edu
GFL Outdoors	www.gfloutdoors.com
Dlium	www.dlium.com
The European Palm Society	www.palmsociety.org
Grow Plants	www.growplants.org
Calyx Flowers	www.calyxflowers.com
Mt. Cuba Center	mtcubacenter.org
Balkan Ecology Project	www.balkep.org
Dear Plants	www.dearplants.com
Garden Oracle - Drought and Heat Tolerant Gardening	gardenoracle.com
Darwin Perennials	www.darwinperennials.com
Woodland Trust	www.woodlandtrust.org.uk

Moores Valley Nurseries	www.mooresvalleynurseries.co.nz
The American Rhododendron Society	www.rhododendron.org
Millais Nurseries - Rhododendron Specialists	www.rhododendrons.co.uk
Oregon State University, Dept. of Horticulture - Landscape Plants	landscapeplants.oregonstate.edu
World Plants	www.worldplants.ca
Benara Nurseries	www.benaranurseries.com
Australian Home Design Directory	www.homedesigndirectory.com.au
Florida Native Plant Society	www.fnps.org
Garden Decorexpro.com	garden.decorexpro.com
Alpine Garden Society Plant Encyclopedia	encyclopaedia.alpinegardensociety.net
Tennessee-Kentucky Plant Atlas	tennessee-kentucky.plantatlas.usf.edu
Rare Exotic Seeds	www.rarexoticseeds.com/en
Lilies by Blewden	www.lilies.co.nz
India Biodiversity Portal	indiabiodiversity.org
Farming Reader	farmingreader.com
Australian Native Plants	www.australianplants.com
Royal New Zealand Institute of Horticulture	www.rnzih.org.nz
University of Washington	depts.washington.edu/propplnt/Plants
Keeping It Green Nursery	www.keepingitgreennursery.com
Nurseries Online Australia	www.nurseriesonline.com.au
Gardener's Path	gardenerspath.com
North Dakota State University - Tree Information Center	www.ag.ndsu.edu/trees
Hayloft	hayloft.co.uk
Greening the Canadian Landscape	www.greeningcanadianlandscape.ca/tree-species-calculator/tree-species-calculator-list
Department of Primary Industries - New South Wales	www.dpi.nsw.gov.au
Bruns Pflanzen	online.bruns.de/en-us
Arbor Day Foundation	www.arborday.org
One Nature Plant Nursery	www.onenaturenursery.com
Nature Hills	www.naturehills.com
Week &	www.weekand.com/home-garden
Horticulture Magazine	horticulture.co.uk
Plant File	www.plantfileonline.net
Plant Selector - Botanic Gardens of South Australia	plantselector.botanicgardens.sa.gov.au
Rock Garden Plants Database	flora.kadel.cz
Alabama Plant Atlas	floraofalabama.org
Succulent Guide	succulent.guide
Aussie Green Thumb	aussiegreenthumb.com
TUIN Seizoen	tuinseizoen.com
Doon Gardening Society	doongardeningsociety.yolasite.com
What Grows There	www.whatgrowsthere.com/grow
Planting Man	plantingman.com
The Garden Helper	www.thegardenhelper.com
Seasonal Gardening	www.seasonalgardening.co.uk
An Eco-sustainable World	antropocene.it/en

TreeLib	treelib.ca
Things You Must Know - Plants Encyclopedia	www.things-you-must-know.com/plant
Lady Bird Johnson Wildflower Center - University of Texas at Austin	www.wildflower.org/plants
Chateau Perouse Botanic Garden - Database	www.chateau-perouse.com
Plantmark - Wholsesale Nurseries	www.plantmark.com.au
Botanic Gardens and Parks Authority - Government of Western Australia	www.bgpa.wa.gov.au/about-us/information/our-plants
JSTOR Global Plants	plants.jstor.org
University of Delaware Botanic Gardens	canr.udel.edu/udbg
Jardineria On (Gardening On)	www.jardineriaon.com/en
Florabase - by Western Australian Herbarium	florabase.dpaw.wa.gov.au
Hardy Eucalyptus	www.hardy-eucalyptus.com
WoodiWild	woodiwild.org
Tree Project	treeproject.org.au
Monaco Nature Encyclopedia	www.monaconatureencyclopedia.com
Hunker	www.hunker.com
Gardeners HQ	www.gardenershq.com
Theodore Payne Foundation for Wild Flowers & Native Plants	theodorepayne.org/nativeplantdatabase
A Guide to the Tasmanian Flora	tasmanianflora.com
Housing.com	housing.com/news/category/lifestyle/gardening/
Far Reaches Farm	www.farreachesfarm.com
Botanically Inclined (Seeds Shop)	botanicallyinclined.org
Prof-Seeds	prof-seeds.com
Australasian Plant Society (UK)	www.anzplantsoc.org.uk
mygardenlife.com	mygardenlife.com
Waterwise Garden Planner - for Southern California	waterwisegardenplanner.org
Irish Garden Plant Society	irishgardenplantsociety.com
Online Plant Guide	www.onlineplantguide.com
Burncoose Nurseries	www.burncoose.co.uk
Ace Gardener - Landscaping & Gardening	acegardener.ca
