# Supplementary material for: Historical frequency of plants in nursery catalogues predicts likelihood of naturalization in ornamental species
Source: Ecol Appl. 2025 May 11;35(3):e70023. doi: 10.1002/eap.70023 (PMC12066803; doi:10.1002/eap.70023)
Supplement: Supplementary file 3 — Appendix S3. [file EAP-35-e70023-s004.pdf]

Historical frequency of plants in nursery catalogues predicts likelihood of naturalization in ornamental species. Thomas N. Dawes, Jennifer L. Bufford, and Philip E. Hulme. *Ecological Applications*.

<u>Appendix S3</u>

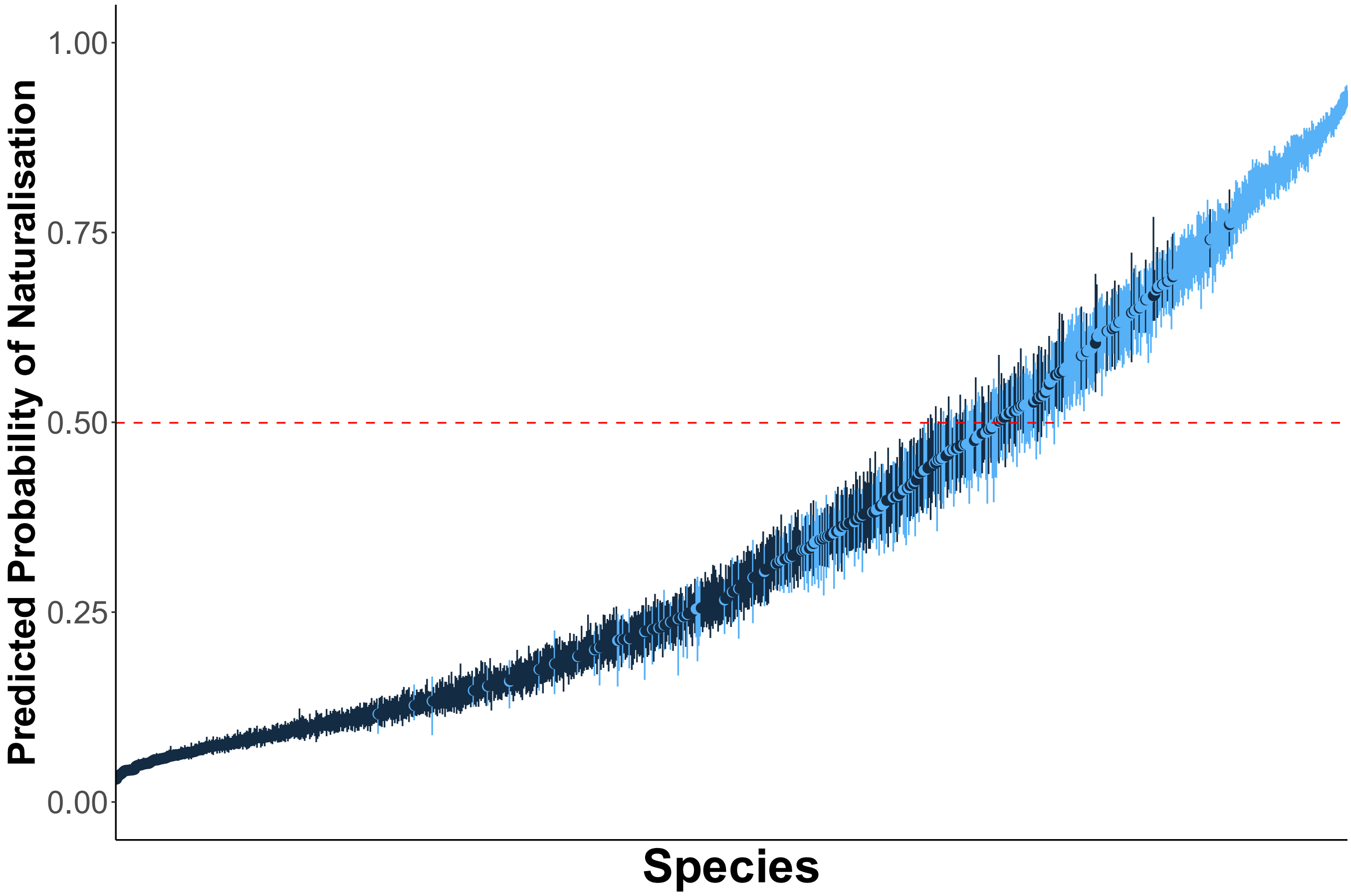

Appendix S3: Figure S1 – A plot showing the mean and quartile ranges of the predicted values of naturalisation for each of the 958 species across 100 model runs. Species are ordered by mean predicted value. Species coloured pale blue are naturalised, whilst species coloured black are non-naturalised. The dashed red line shows the MCC threshold (0.5) for determining how species are classified. Thus, species coloured blue with a mean predicted value above the line and coloured black below the line are correctly classified by the model. Conversely, species coloured black above the line or coloured blue below the line are incorrectly classified by the model.

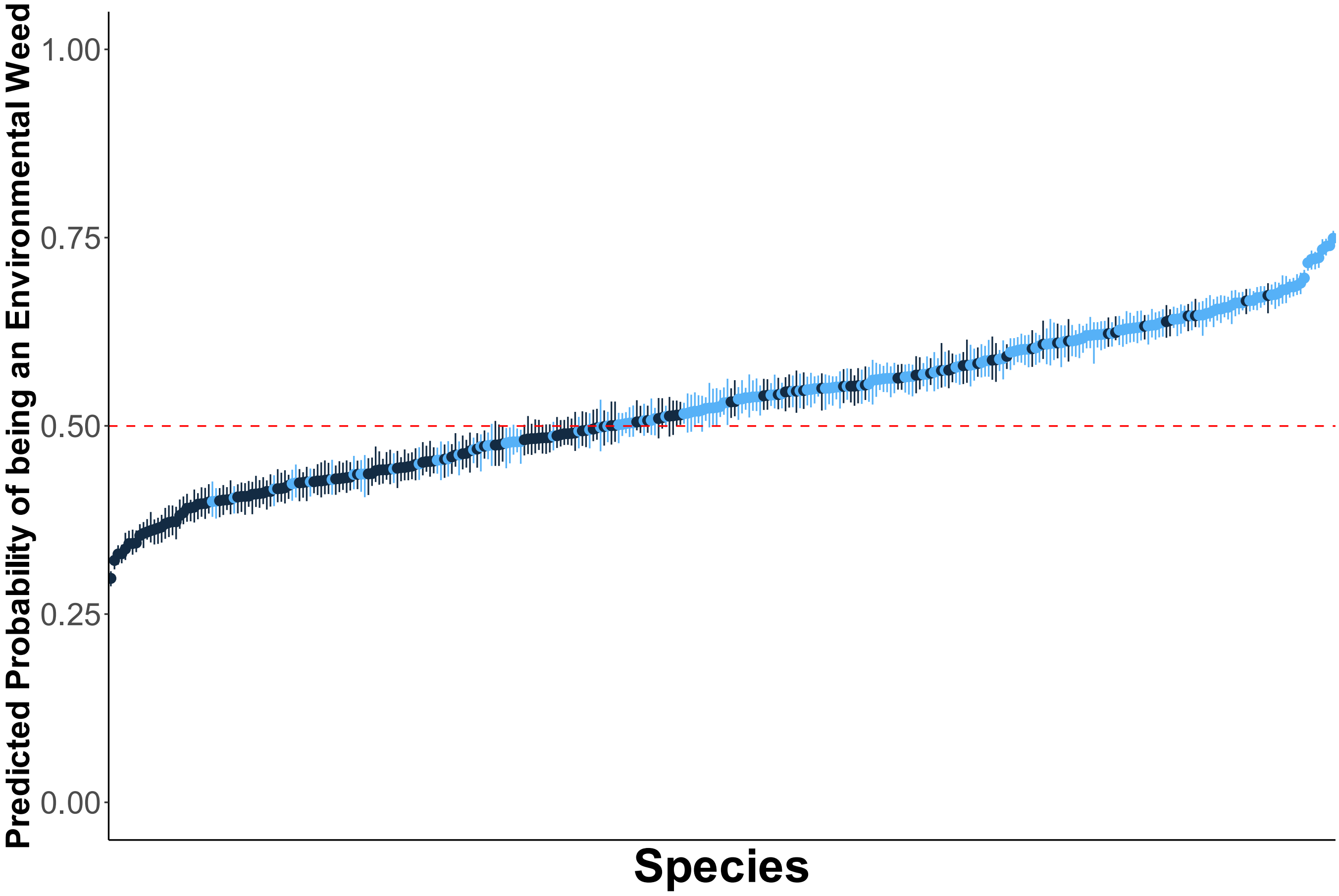

Appendix S3: Figure S2 – A plot showing the mean and quartile ranges of the predicted values of being classified as an invasive environmental weed for each of the 337 species across 100 model runs. Species are ordered by mean predicted value. Species coloured pale blue are classified as environmental weeds, whilst species coloured black are not. The dashed red line shows the MCC threshold (0.5) for determining how species are classified. Thus, species coloured blue with a mean predicted value above the line are correctly classified by the model, species coloured black with a mean predicted value below the line are also correctly classified. Conversely, species coloured black above the line or coloured blue below the line are incorrectly classified by the model.
