# Supplementary material for: Historical frequency of plants in nursery catalogues predicts likelihood of naturalization in ornamental species
Source: Ecol Appl. 2025 May 11;35(3):e70023. doi: 10.1002/eap.70023 (PMC12066803; doi:10.1002/eap.70023)
Supplement: Supplementary file 4 — Appendix S4. [file EAP-35-e70023-s005.pdf]

**Historical frequency of plants in nursery catalogues predicts likelihood of naturalization in ornamental species.** Thomas N. Dawes, Jennifer L. Bufford, and Philip E. Hulme. *Ecological Applications*.

## Appendix S4

Two related tables listing the species incorrectly predicted by the mean of the naturalisation model runs. **Appendix S4: Table S1** shows the non-naturalised species that were incorrectly predicted as naturalised by our mean naturalisation model. Species are ordered in descending order of mean predicted probability of naturalisation across the 100 model runs, thus placing the most misclassified species at the top. The top 5 misclassified species are highlighted pale yellow. **Appendix S4: Table S2** shows the naturalised species incorrectly predicted as non-naturalised by the model. These are also ordered by mean predicted probability of naturalisation, but in this case in ascending order to also have the most misclassified species at the top. The top 5 misclassified species are also highlighted pale yellow.

### Appendix S4: Table S1:

| Species | Family | Mean Predicted Probability of Naturalisation |
|---|---|---|
| *Campsis grandiflora* | Bignoniaceae | 0.77 |
| *Prunus glandulosa* | Rosaceae | 0.75 |
| *Picea smithiana* | Pinaceae | 0.71 |
| *Rosa banksiae* | Rosaceae | 0.70 |
| *Wisteria floribunda* | Fabaceae | 0.69 |
| *Campsis radicans* | Bignoniaceae | 0.69 |
| *Melaleuca lophantha* | Myrtaceae | 0.68 |
| *Hydrangea paniculata* | Hydrangeaceae | 0.67 |
| *Myrtus communis* | Myrtaceae | 0.67 |
| *Agapanthus africanus* | Amaryllidaceae | 0.66 |
| *Eugenia uniflora* | Myrtaceae | 0.66 |
| *Eucalyptus amygdalina* | Myrtaceae | 0.65 |
| *Cupressus torulosa* | Cupressaceae | 0.65 |
| *Chamaecyparis pisifera* | Cupressaceae | 0.64 |
| *Corymbia citriodora* | Myrtaceae | 0.63 |
| *Lonicera hildebrandiana* | Caprifoliaceae | 0.63 |
| *Moraea aristata* | Iridaceae | 0.62 |
| *Tradescantia zebrina* | Commelinaceae | 0.62 |
| *Prunus salicina* | Rosaceae | 0.60 |
| *Iris pumila* | Iridaceae | 0.60 |
| *Elaeagnus pungens* | Elaeagnaceae | 0.59 |
| *Ficus elastica* | Moraceae | 0.58 |
| *Eucalyptus camaldulensis* | Myrtaceae | 0.58 |
| *Rhododendron indicum* | Ericaceae | 0.57 |
| *Mespilus germanica* | Rosaceae | 0.57 |
| *Drepanostachyum falcatum* | Poaceae | 0.57 |
| *Ligustrum japonicum* | Oleaceae | 0.57 |
| *Platycladus orientalis* | Cupressaceae | 0.55 |

| | | |
|---|---|---|
| *Ulmus minor* | Ulmaceae | 0.55 |
| *Allium schoenoprasum* | Amaryllidaceae | 0.55 |
| *Solanum seaforthianum* | Solanaceae | 0.54 |
| *Acer platanoides* | Sapindaceae | 0.54 |
| *Agrostis perennans* | Poaceae | 0.53 |
| *Wisteria frutescens* | Fabaceae | 0.53 |
| *Pinus lambertiana* | Pinaceae | 0.53 |
| *Erica ventricosa* | Ericaceae | 0.53 |
| *Iris ensata* | Iridaceae | 0.53 |
| *Acacia buxifolia* | Fabaceae | 0.52 |
| *Rubus armeniacus* | Rosaceae | 0.52 |
| *Pyracantha coccinea* | Rosaceae | 0.52 |
| *Abutilon x hybridum* | Malvaceae | 0.52 |
| *Stipa neaei* | Poaceae | 0.52 |
| *Lilium auratum* | Liliaceae | 0.51 |
| *Alkekengi officinarum* | Solanaceae | 0.51 |
| *Abies procera* | Pinaceae | 0.51 |
| *Jasminum grandiflorum* | Oleaceae | 0.50 |

**Appendix S4: Table S2:**

| **Species** | **Family** | **Mean Predicted Probability of Naturalisation** |
|---|---|---|
| *Ricinus communis* | Euphorbiaceae | 0.11 |
| *Erica caffra* | Ericaceae | 0.13 |
| *Cotoneaster bullatus* | Rosaceae | 0.13 |
| *Genista tinctoria* | Fabaceae | 0.14 |
| *Rubus phoenicolasius* | Rosaceae | 0.15 |
| *Rosa gallica* | Rosaceae | 0.16 |
| *Syzygium australe* | Myrtaceae | 0.17 |
| *Hakea drupacea* | Proteaceae | 0.18 |
| *Polypodium vulgare* | Polypodiaceae | 0.19 |
| *Frangula purshiana* | Rhamnaceae | 0.19 |
| *Acacia mearnsii* | Fabaceae | 0.19 |
| *Bambusa oldhamii* | Poaceae | 0.20 |
| *Rosa rubiginosa* | Rosaceae | 0.21 |
| *Psidium guajava* | Myrtaceae | 0.21 |
| *Rubus laciniatus* | Rosaceae | 0.21 |
| *Pinus taeda* | Pinaceae | 0.21 |
| *Celastrus orbiculatus* | Celastraceae | 0.22 |
| *Prunus mahaleb* | Rosaceae | 0.22 |
| *Thymus pulegioides* | Lamiaceae | 0.23 |
| *Calicotome spinosa* | Fabaceae | 0.23 |
| *Crocosmia x crocosmiiflora* | Iridaceae | 0.23 |

| | | |
|---|---|---|
| *Ajuga reptans* | Lamiaceae | 0.23 |
| *Carpobrotus edulis* | Aizoaceae | 0.24 |
| *Symphyotrichum novae-angliae* | Asteraceae | 0.24 |
| *Rubus pensilvanicus* | Rosaceae | 0.25 |
| *Abies grandis* | Pinaceae | 0.25 |
| *Pinus patula* | Pinaceae | 0.25 |
| *Pinus monticola* | Pinaceae | 0.26 |
| *Crataegus monogyna* | Rosaceae | 0.27 |
| *Prunus cerasus* | Rosaceae | 0.27 |
| *Ochna serrulata* | Ochnaceae | 0.29 |
| *Arum italicum* | Araceae | 0.30 |
| *Elodea densa* | Hydrocharitaceae | 0.30 |
| *Clematis terniflora* | Ranunculaceae | 0.31 |
| *Erica baccans* | Ericaceae | 0.31 |
| *Tradescantia fluminensis* | Commelinaceae | 0.31 |
| *Photinia bodinieri* | Rosaceae | 0.32 |
| *Osmunda regalis* | Osmundaceae | 0.32 |
| *Sorbaria tomentosa* | Rosaceae | 0.33 |
| *Asphodelus fistulosus* | Asphodelaceae | 0.33 |
| *Populus tremula* | Salicaceae | 0.33 |
| *Eucalyptus cinerea* | Myrtaceae | 0.33 |
| *Senna multiglandulosa* | Fabaceae | 0.33 |
| *Rhamnus alaternus* | Rhamnaceae | 0.33 |
| *Sambucus nigra* | Viburnaceae | 0.33 |
| *Lupinus polyphyllus* | Fabaceae | 0.33 |
| *Cotoneaster glaucophyllus* | Rosaceae | 0.33 |
| *Pleioblastus viridistriatus* | Poaceae | 0.34 |
| *Cotoneaster horizontalis* | Rosaceae | 0.34 |
| *Tetrapanax papyrifer* | Araliaceae | 0.34 |
| *Panicum virgatum* | Poaceae | 0.34 |
| *Dryopteris filix-mas* | Dryopteridaceae | 0.34 |
| *Cotoneaster coriaceus* | Rosaceae | 0.34 |
| *Rubus flagellaris* | Rosaceae | 0.35 |
| *Eucalyptus eugenioides* | Myrtaceae | 0.35 |
| *Lonicera japonica* | Caprifoliaceae | 0.35 |
| *Roldana petasitis* | Asteraceae | 0.36 |
| *Aloiampelos ciliaris* | Asphodelaceae | 0.36 |
| *Eucalyptus tereticornis* | Myrtaceae | 0.36 |
| *Dracunculus vulgaris* | Araceae | 0.37 |
| *Ribes aureum* | Grossulariaceae | 0.37 |
| *Salix eleagnos* | Salicaceae | 0.38 |
| *Cestrum nocturnum* | Solanaceae | 0.38 |
| *Eucalyptus robusta* | Myrtaceae | 0.38 |
| *Symphyotrichum novi-belgii* | Asteraceae | 0.38 |
| *Cardiocrinum giganteum* | Liliaceae | 0.39 |
| *Passiflora tripartita* | Passifloraceae | 0.39 |
| *Anemonoides nemorosa* | Ranunculaceae | 0.39 |

| | | |
|---|---|---|
| *Hakea sericea* | Proteaceae | 0.39 |
| *Humulus lupulus* | Cannabaceae | 0.39 |
| *Sambucus racemosa* | Viburnaceae | 0.39 |
| *Juglans ailantifolia* | Juglandaceae | 0.40 |
| *Crassula spathulata* | Crassulaceae | 0.40 |
| *Pinus halepensis* | Pinaceae | 0.40 |
| *Ulmus x hollandica* | Ulmaceae | 0.40 |
| *Erica vagans* | Ericaceae | 0.41 |
| *Selaginella kraussiana* | Selaginellaceae | 0.42 |
| *Phyla nodiflora* | Verbenaceae | 0.42 |
| *Jasminum beesianum* | Oleaceae | 0.43 |
| *Salix viminalis* | Salicaceae | 0.43 |
| *Ipomoea alba* | Convolvulaceae | 0.44 |
| *Pinus nigra* | Pinaceae | 0.44 |
| *Passiflora caerulea* | Passifloraceae | 0.44 |
| *Festuca rubra* | Poaceae | 0.44 |
| *Hakea salicifolia* | Proteaceae | 0.45 |
| *Cotyledon orbiculata* | Crassulaceae | 0.45 |
| *Vinca major* | Apocynaceae | 0.45 |
| *Picea sitchensis* | Pinaceae | 0.45 |
| *Catharanthus roseus* | Apocynaceae | 0.45 |
| *Tropaeolum pentaphyllum* | Tropaeolaceae | 0.46 |
| *Cestrum fasciculatum* | Solanaceae | 0.46 |
| *Dipogon lignosus* | Fabaceae | 0.46 |
| *Watsonia marginata* | Iridaceae | 0.46 |
| *Spartium junceum* | Fabaceae | 0.46 |
| *Plectranthus ecklonii* | Lamiaceae | 0.46 |
| *Erica cinerea* | Ericaceae | 0.46 |
| *Hylotelephium spectabile* | Crassulaceae | 0.46 |
| *Nephrolepis cordifolia* | Nephrolepidaceae | 0.47 |
| *Hordeum jubatum* | Poaceae | 0.47 |
| *Pinus contorta* | Pinaceae | 0.47 |
| *Zantedeschia albomaculata* | Araceae | 0.47 |
| *Dactylis glomerata* | Poaceae | 0.48 |
| *Polygala myrtifolia* | Polygalaceae | 0.48 |
| *Passiflora mixta* | Passifloraceae | 0.48 |
| *Podalyria sericea* | Fabaceae | 0.48 |
| *Populus deltoides* | Salicaceae | 0.48 |
| *Aloe arborescens* | Asphodelaceae | 0.48 |
| *Sedum dasyphyllum* | Crassulaceae | 0.48 |
| *Sedum album* | Crassulaceae | 0.48 |
| *Rubus fruticosus* | Rosaceae | 0.49 |
| *Polygala virgata* | Polygalaceae | 0.49 |
| *Eriocapitella x hybrida* | Ranunculaceae | 0.50 |
| *Nuphar lutea* | Nymphaeaceae | 0.50 |
| *Oxylobium lanceolatum* | Fabaceae | 0.50 |
