# Supplementary material for: Historical frequency of plants in nursery catalogues predicts likelihood of naturalization in ornamental species
Source: Ecol Appl. 2025 May 11;35(3):e70023. doi: 10.1002/eap.70023 (PMC12066803; doi:10.1002/eap.70023)
Supplement: Supplementary file 5 — Appendix S5. [file EAP-35-e70023-s001.pdf]

Historical frequency of plants in nursery catalogues predicts likelihood of naturalization in ornamental species. Thomas N. Dawes, Jennifer L. Bufford, and Philip E. Hulme. *Ecological Applications*.

Appendix S5

Two related tables listing the species incorrectly predicted by the mean invasiveness model run. **Appendix S5: Table S1** shows the non-invasive species that were incorrectly predicted as invasive by our mean invasiveness model. Species are ordered in descending order of mean predicted probability of being classed as invasive across the 100 model runs, thus placing the most misclassified species at the top. **Appendix S5: Table S2** shows the invasive species incorrectly predicted as non-invasive by the model. These are also ordered by mean predicted probability of being classed as invasive, but in this case in ascending order to also have the most misclassified species at the top.

Appendix S5: Table S1:

Species	Family	Mean Predicted Probability of Invasiveness
Cupressus sempervirens	Cupressaceae	0.67
Juglans regia	Juglandaceae	0.67
Viburnum plicatum	Viburnaceae	0.65
Acacia baileyana	Fabaceae	0.65
Picea abies	Pinaceae	0.64
Pyrus communis	Rosaceae	0.64
Ficus pumila	Moraceae	0.63
Viburnum tinus	Viburnaceae	0.63
Abies nordmanniana	Pinaceae	0.62
Jasminum officinale	Oleaceae	0.62
Acer negundo	Sapindaceae	0.61
Polygala virgata	Polygalaceae	0.61
Solanum betaceum	Solanaceae	0.60
Buddleja globosa	Scrophulariaceae	0.59
Hesperocyparis lusitanica	Cupressaceae	0.59
Erythrina crista-galli	Fabaceae	0.59
Erica cinerea	Ericaceae	0.58
Berberis vulgaris	Berberidaceae	0.58
Phyllostachys nigra	Poaceae	0.58
Prunus lusitanica	Rosaceae	0.58
Cotoneaster integrifolius	Rosaceae	0.58
Ribes nigrum	Grossulariaceae	0.57
Passiflora mixta	Passifloraceae	0.57
Bambusa multiplex	Poaceae	0.57
Catharanthus roseus	Apocynaceae	0.57
Podranea ricasoliana	Bignoniaceae	0.56
Miscanthus sinensis	Poaceae	0.56
Parthenocissus quinquefolia	Vitaceae	0.56
Lonicera ligustrina	Caprifoliaceae	0.55
Spartium junceum	Fabaceae	0.55

Ligustrum vulgare	Oleaceae	0.55
Rosa multiflora	Rosaceae	0.55
Colocasia esculenta	Araceae	0.55
Asparagus officinalis	Asparagaceae	0.55
Laburnum anagyroidis	Fabaceae	0.55
Ribes rubrum	Grossulariaceae	0.55
Aloe arborescens	Asphodelaceae	0.55
Campsis x tagliabuana	Bignoniaceae	0.54
Abies grandis	Pinaceae	0.54
Cordyline fruticosa	Asparagaceae	0.54
Cytisus multiflorus	Fabaceae	0.54
Brugmansia sanguinea	Solanaceae	0.53
Genista stenopetala	Fabaceae	0.52
Lycianthes rantonnetii	Solanaceae	0.51
Cotoneaster pannosus	Rosaceae	0.51
Populus nigra	Salicaceae	0.51
Buddleja salviifolia	Scrophulariaceae	0.51
Cestrum fasciculatum	Solanaceae	0.51
Hedera canariensis	Araliaceae	0.51
Pleioblastus viridistriatus	Poaceae	0.51
Picea sitchensis	Pinaceae	0.51
Vinca minor	Apocynaceae	0.51
Impatiens walleriana	Balsaminaceae	0.50
Berberis aquifolium	Berberidaceae	0.50

Appendix S5: Table S2:

**Species**	**Family**	**Mean Predicted Probability of Invasiveness**
Sedum acre	Crassulaceae	0.40
Rubus fruticosus	Rosaceae	0.40
Rosa rubiginosa	Rosaceae	0.40
Crocosmia x crocosmiiflora	Iridaceae	0.41
Rubus phoenicolasius	Rosaceae	0.42
Festuca rubra	Poaceae	0.42
Osmunda regalis	Osmundaceae	0.43
Selaginella kraussiana	Selaginellaceae	0.43
Eucalyptus delegatensis	Myrtaceae	0.43
Ajuga reptans	Lamiaceae	0.44
Carpobrotus edulis	Aizoaceae	0.44
Arum italicum	Araceae	0.45
Iris pseudacorus	Iridaceae	0.45
Elodea densa	Hydrocharitaceae	0.45
Cestrum nocturnum	Solanaceae	0.45
Lythrum salicaria	Lythraceae	0.46
Lupinus polyphyllus	Fabaceae	0.46
Acacia melanoxylon	Fabaceae	0.46

Phragmites australis	Poaceae	0.47
Kalanchoe pinnata	Crassulaceae	0.47
Ulmus x hollandica	Ulmaceae	0.47
Roldana petasitis	Asteraceae	0.47
Acacia mearnsii	Fabaceae	0.48
Ageratina riparia	Asteraceae	0.48
Asphodelus fistulosus	Asphodelaceae	0.48
Syzygium australe	Myrtaceae	0.48
Acacia dealbata	Fabaceae	0.49
Lilium lancifolium	Liliaceae	0.49
Arundo donax	Poaceae	0.49
Iris foetidissima	Iridaceae	0.50
Celastrus orbiculatus	Celastraceae	0.50
Furcraea foetida	Asparagaceae	0.50
